# α-Synuclein Aggregated with Tau and β-Amyloid in Human Platelets from Healthy Subjects: Correlation with Physical Exercise

**DOI:** 10.3389/fnagi.2018.00017

**Published:** 2018-01-30

**Authors:** Simona Daniele, Deborah Pietrobono, Jonathan Fusi, Annalisa Lo Gerfo, Eugenio Cerri, Lucia Chico, Caterina Iofrida, Lucia Petrozzi, Filippo Baldacci, Chiara Giacomelli, Fabio Galetta, Gabriele Siciliano, Ubaldo Bonuccelli, Maria L. Trincavelli, Ferdinando Franzoni, Claudia Martini

**Affiliations:** ^1^Department of Pharmacy, University of Pisa, Pisa, Italy; ^2^Department of Clinical and Experimental Medicine, University of Pisa, Pisa, Italy

**Keywords:** protein misfolding, α-synuclein, β-amyloid, tau, α-synuclein heterocomplexes, platelets, antioxidant capability, physical exercise

## Abstract

The loss of protein homeostasis that has been associated with aging leads to altered levels and conformational instability of proteins, which tend to form toxic aggregates. In particular, brain aging presents characteristic patterns of misfolded oligomers, primarily constituted of β-amyloid (Aβ), tau, and α-synuclein (α-syn), which can accumulate in neuronal membranes or extracellular compartments. Such aging-related proteins can also reach peripheral compartments, thus suggesting the possibility to monitor their accumulation in more accessible fluids. In this respect, we have demonstrated that α-syn forms detectable hetero-aggregates with Aβ or tau in red blood cells (RBCs) of healthy subjects. In particular, α-syn levels and its heteromeric interactions are modulated by plasma antioxidant capability (AOC), which increases in turn with physical activity. In order to understand if a specific distribution of misfolded proteins can occur in other blood cells, a cohort of human subjects was enrolled to establish a correlation among AOC, the level of physical exercise and the concentrations of aging-related proteins in platelets. The healthy subjects were divided depending on their level of physical exercise (i.e., athletes and sedentary subjects) and their age (young and older subjects). Herein, aging-related proteins (i.e., α-syn, tau and Aβ) were confirmed to be present in human platelets. Among such proteins, platelet tau concentration was demonstrated to decrease in athletes, while α-syn and Aβ did not correlate with physical exercise. For the first time, α-syn was shown to directly interact with Aβ and tau in platelets, forming detectable hetero-complexes. Interestingly, α-syn interaction with tau was inversely related to plasma AOC and to the level of physical activity. These results suggested that α-syn heterocomplexes, particularly with tau, could represent novel indicators to monitor aging-related proteins in platelets.

## Introduction

Aging is characterized by a gradual decline of the protein homeostasis (proteostasis) network, which affects the levels and conformational stability of proteins (Díaz-Villanueva et al., 2015; Labbadia and Morimoto, 2015), causing the formation of misfolded and toxic aggregates (Lindner and Demarez, 2009; Morimoto and Cuervo, 2014; Tan et al., 2014; Labbadia and Morimoto, 2015), and ultimately cell death.

Misfolded and oligomeric proteins appear to spread through the brain in characteristic, with robust evidence for the increase of beta amyloid_1-42_ (Aβ), tau, and α-synuclein (α-syn) (Jucker and Walker, 2013; Goedert, 2015; Giacomelli et al., 2017). In addition, the occurrence of hybrid oligomers (“heteroaggregates”) has been shown in both patients’ brains and cellular models (Parnetti et al., 2013; Sengupta et al., 2015; Andersen et al., 2017; Daniele et al., 2017; Giacomelli et al., 2017). In this respect, the levels of the so called “aging-related proteins” have been monitored recently in human red blood cells (RBCs), where α-syn has been shown to interact with Aβ or tau healthy subjects (Daniele et al., 2017).

Accumulation of toxic, possibly infectious, protein aggregates induces a cascade of events, such as excessive inflammation, the production of ROS, apoptosis and neuronal loss (Hinault et al., 2006). Moreover, proteinopathy and oxidative stress, together with mitochondrial dysfunction, represent “interacting damage pathways” that culminate in cellular degeneration. In fact, ROS have been demonstrate to enhance the aggregation, of neurodegeneration-related proteins that, in turn, may interact with transition metals or other components to generate further ROS (Snead and Eliezer, 2014; Xiang et al., 2015).

Regular physical activity has been emerging as a pivotal factor in counteracting cellular oxidative stress, by exerting anti-inflammatory actions (Sallam and Laher, 2016), up-regulating the enzymatic antioxidant system (Voss et al., 2013; Radak et al., 2016), but also increasing neurogenesis and proteolytic degradation of misfolded proteins (Johnson and Mitchell, 2003; Lazarov et al., 2005; Radak et al., 2013). Consequently, it is not surprisingly that exercise has been shown also to impact protein oligomerization; for example, running has been related to a reduced Aβ deposition and tau phosphorylation in mouse brains (Brown et al., 2013; Baldacci et al., 2016; Tapia-Rojas et al., 2016; Koo et al., 2017). Recently, data on such beneficial effects have been emerging in human subjects at a peripheral level, too (DeMattos et al., 2001; Eisele et al., 2010; Brown et al., 2013). In fact, protein accumulation and aggregation has been demonstrated to reach the cerebrospinal and peripheral fluids, too (Tokuda et al., 2010). Therefore, biological changes and putative peripheral biomarkers can be exploited in tissue other than the brain.

In this respect, we have demonstrated that aging-related proteins and in particular α-syn heteromeric interactions with Aβ or tau can be modulated by both oxidative stress and physical exercise (Daniele et al., 2017).

In order to understand if a specific distribution of misfolded proteins can occur in other blood cells, herein the presence and levels of aging-related proteins were investigated in platelets isolated from human subjects, grouped by their level of physical exercise (i.e., athletes and sedentary subjects) and their age (young and older subjects). Specifically, α-syn heterocomplexes with Aβ or tau were measured to unveil their presence and putative pathogenic role in platelets (Veitinger et al., 2014). Moreover, plasma AOC, the primary marker of oxidative stress in aging-related pathologies (Pandey and Rizvi, 2010), was quantified in the same cohort and correlated to the grade of physical exercise.

## Materials and Methods

### Study Population and Setting of the Study

Endurance athletes (*N* = 45, ATHL, mean age 45.2 ± 13.0 years) and age-sex-matched sedentary volunteers (*N* = 58, SED, mean age 47.6 ± 14.4 years) were recruited from the Sport Medicine Unit of the Department of Clinical and Experimental Medicine of the University of Pisa (**Table 1**).

**Table 1:** Descriptive analysis of the total population and of the subgroups.

	Number of subjects (*N*)	Age (y)	BMI	Heart rate	Physical activity level
Young subjects	55	36.1 ± 8.8	23.2 ± 1.9	55.7 ± 3.8	9.94 ± 3.92
Older subjects	48	58.4 ± 7.4	23.7 ± 2.1	59.3 ± 5.8	8.90 ± 3.20
ATHL	45	45.2 ± 13.0	23.8 ± 1.9	50.6 ± 3.8	13.2 ± 2.2^∗^
SED	58	47.6 ± 14.4	24.0 ± 1.6	62.3 ± 5.2	6.61 ± 0.64

*The data are the mean ± SD. BMI, body mass index; ATHL, athletes, SED, sedentary. Differences between groups (i.e., young vs. older and ATHL vs. SED) were evaluated by One-way ANOVA followed by a Kruskal–Wallis *post hoc* test. *P*-values were adjusted with Sidak’s multiple comparison test. ^∗^*P* < 0.001 ATHL vs. SED subjects.*

Major inclusion criteria were as followed: total plasma cholesterol ranging from 3.1 to 5.8 mmol/L, HDL cholesterol from 0.67 to 1.9 mmol/L, plasma triglycerides from 0.34 to 1.7 mmol/L, body mass index lower than 30 kg/m^2^, diastolic arterial blood pressure lower than 90 mmHg and systolic arterial blood pressure lower than 140 mmHg (Daniele et al., 2017). Subjects were excluded if they had smoking habits or received drug treatments within the previous 2 months (Daniele et al., 2017).

Aerobic fitness was evaluated with a maximal graded cycle ergometry test performed by a cardiologist blinded to the other data (Whaley et al., 2006). Blood pressure and the rate of perceived exertion (RPE) were assessed at the end of each step. The 15-point Borg RPE scale (Borg, 1982; Hamer and Slocombe, 1997) was used to evaluate the level of intensity for each participant (Daniele et al., 2017). The scale ranges from 6 to 20, with 6 corresponding to no exertion at all, 7.5 to extremely light, 9 to very light, 11 to light, 13 to somewhat hard, 15 to hard, 17 to very hard, 19 to extremely hard, and 20 to maximal exertion.

The enrolled healthy subjects were divided into younger (≤50 years), older (>50 years), athletes (ATHL) and sedentary (SED) subgroups (**Table 1** and see “Results” section). The time period between the last exercise bout and blood sampling was at least 48 h.

This study was approved by the Ethics Committee of the Great North West Area of Tuscany (271/2014 to FF) and it was carried out in accordance with the Declaration of Helsinki. All subjects gave informed consent to participate in the study. Fully informed consent was obtained from each subject entering the study (Daniele et al., 2017).

### Plasma and Platelet Collection

Whole blood from healthy volunteers was collected into a tube containing EDTA as an anticoagulant. The plasma was separated by centrifugation (200 × *g* at 4°C for 10 min), and then stored at -80°C until use.

To obtain platelets, platelet-rich plasma was collected and centrifuged at 7000 × *g* for 10 min at 4°C. The platelet pellets were washed with 0,83% NH_4_Cl and centrifuged at 1500 × *g*. Then, the pellet was washed with 3 ml of PBS, centrifuged and frozen at -20°C.

### Co-immunoprecipitation and Western Blotting Analysis

The α-syn, tau and Aβ expression was assessed as described previously (Daniele et al., 2017). In brief, platelets (1,8 mg of proteins) were lysed with RIPA buffer (Costa et al., 2013) and then resolved by SDS-PAGE (8.5%). Platelets were incubated with primary antibodies to α-syn (α/β-synuclein N-19, SC-7012, Santa Cruz Biotechnology), tau (H-150 SC-5587, Santa Cruz Biotechnology) or Aβ (β-amyloid H-43 SC-9129, Santa Cruz Biotechnology) overnight at 4°C (Daniele et al., 2017). The primary antibodies were detected using peroxidase- conjugated secondary antibodies and a chemioluminescent substrate (ECL, Perkin Elmer).

To verify α-syn interaction with tau or Aβ, a co-immunoprecipitation assay was employed (Costa et al., 2013; Daniele et al., 2017). Briefly, 0.5 mg of platelet lysate proteins was probed overnight under constant rotation with an anti-α-syn antibody (5 μg/sample), and then immunoprecipitated with protein A-Sepharose. After extensive washing, the immunocomplexes were re-suspended in Laemmli solution, resolved by SDS-PAGE and probed overnight with primary antibodies to α-syn (input), tau or Aβ as above described (Daniele et al., 2017).

### Detection of Total α-synuclein

Total α-syn was detected in platelets, as previously described (Foulds et al., 2011; Daniele et al., 2017). In brief, wells were coated using a α-syn full length antibody (sc-10717, Santa Cruz Biotechnology), and non-specific sites were blocked using Bovine Serum Albumine (BSA). Platelet samples (32 μg proteins/100 μl) were added to the wells for 2 h at 25°C. In parallel, recombinant aliquots of α-syn were tested to create a standard curve. After extensive washing, wells were incubated with a different α-syn antibody (Santa Cruz, sc-12767), and subsequently with an anti-mouse-HRP antibody (Daniele et al., 2017). The samples were washed with PBS-T (phosphate buffered saline containing 0.01% Tween 20), and incubated with the enzyme substrate TMB (3,3′,5,5′-tetramethylbenzidine, Thermo Scientific). Absorbance values were read at 450 nm.

### Detection of Total Aβ

Aβ levels in platelets were assessed using an immunoenzymatic assay, as previously described (Pesini et al., 2012; Daniele et al., 2017). A specific anti-Aβ antibody (Santa Cruz, sc-9129) was used for 96-well coating at 4°C. After extensive washing with PBS-T, non-specific sites were blocked with 1% BSA. Platelets (80 μg proteins/100 μl) were added to each well and incubated at 25°C for 1 h. After extensive washing with PBS-T, samples were detected using the polyclonal Aβ antibody (sc-5399, Santa Cruz Biotechnology). The standard curve was achieved using solutions of recombinant human Aβ protein at eight different concentrations (Daniele et al., 2017).

### Detection of Total Tau

Tau levels in platelets were determined using an immunoenzymatic assay, as previously described (Pesini et al., 2012; Daniele et al., 2017). In brief, platelets (32 μg proteins/100 μl) were added to each well pre-coated with a specific anti-tau antibody (Santa Cruz, sc-32274). Then, samples were probed with a different tau antibody (sc-5587, Santa Cruz Biotechnology). In parallel, solutions of recombinant human tau protein were used to obtain a standard curve.

### Detection of α-syn-Aβ Heterocomplexes

The degree of α-syn-Aβ interactions were quantified, as previously described (Daniele et al., 2017). In brief, standard α-syn-Aβ were prepared in 2 mM sodium dodecyl sulfate (SDS) in parafilm-sealed tubes at 37°C for 36 h in an “Eppendorf Thermomixer” with continuous mixing (500 rpm) (Mandal et al., 2006; Daniele et al., 2017).

Platelets (800 μg/sample) were added to wells pre-coated with the β-amyloid H-43 antibody (1:100, sc-9129, Santa Cruz Biotechnology) at 25°C for 2 h. Non-specific sites were blocked with 1% BSA for 30 min at 37°C. The degree of α-syn bound to Aβ was detected using a specific antibody against α-syn (sc-12767, Santa Cruz Biotechnology), and subsequently an appropriate HRP-conjugated antibody (Daniele et al., 2017). Relative concentration of α-syn/Aβ complexes were calculated according to the standard curve obtained in each microplate (Daniele et al., 2017).

### Detection of α-syn-Tau Heterocomplexes

Standard samples of α-syn-tau were prepared in 2 mM SDS (Daniele et al., 2017). Wells were pre-coated with anti-α-syn antibody (1:100, sc-7012, Santa Cruz Biotechnology) and incubated with platelet samples (800 μg/sample) at 25°C for 2 h. Non-specific sites were blocked with 1% BSA for 30 min at 37°C. The wells were probed with a specific tau antibody (sc-5587, Santa Cruz Biotechnology), and subsequently with the appropriate HRP-conjugated antibody (Daniele et al., 2017). Relative concentration of α-syn/tau complexes were calculated according to the standard curve obtained in each microplate (Daniele et al., 2017).

### Total Oxyradical Scavenging Capacity (TOSC) Assay

The plasma antioxidant capability (AOC) was assessed by the TOSC assay, a gas chromatographic assay for determining oxyradical scavenging capacity of biological fluids (Winston et al., 1998; Regoli and Winston, 1999; Daniele et al., 2017). Peroxynitrite was generated from the decomposition of SIN-1 (3-morpholinosyd-nonimine *N*-ethylcarbamide) in the presence of 0.2 mM KMBA [α-cheto-γ-(methylthiol)butyric acid], 100 mM potassium phosphate buffer, pH 7.4, and 0.1 mM DTPA (Diethylene Triamine Penta Acetic Acid), at 35°C. The concentration of SIN-1 was varied to achieve an ethylene yield equivalent to the iron–ascorbate and ABAP systems. Reactions with 0.2 mM KMBA were carried out in 10 ml vials sealed with gas-tight Mininert1 valves (Supelco, Bellefonte, PA, United States) in a final volume of 1 ml. TOSC values were quantified from the equation TOSC = 100 - (SA/CA × 100), where SA and CA, are respectively, the area under the curve (AUC) for sample and control reaction. A TOSC value of 0 corresponds to a sample with no scavenging capacity (Regoli and Winston, 1999; Franzoni et al., 2006; Daniele et al., 2017).

### Statistical Analysis

Data are expressed as mean value ± SD. The population included in this study presented a normal distribution for age. Differences between groups (i.e., young vs. older and ATHL vs. SED) were evaluated by One-way ANOVA followed by a Kruskal–Wallis *post hoc* test. *P*-values were adjusted with Sidak’s multiple comparison test. Correlation between variables was determined by simple linear regression analysis, while covariate analysis was performed by partial correlation matrix. All statistical procedures were performed using the StatView program (Abacus Concepts, Inc., SAS Institute, Cary, NC, United States) (Franzoni et al., 2005; Daniele et al., 2017).

## Results

### Descriptive Statistics

The whole cohort (*N* = 103) was divided in four subgroups (young SED, young ATHL, older SED and older ATHL), whose clinical characteristics are reported in **Table 1**. The mean age of young and older cohort was of 36.1 ± 8.8 and 59.4 ± 7.4, respectively. No significant differences in age, sex and body mass index (BMI) was found between ATHL and SED. As expected, the level of physical activity was significantly higher in the ATHL group than the SED group (*P* < 0.001).

### Expression of α-syn, tau, Aβ Proteins and of α-syn-Tau and α-syn-Aβ Heterocomplexes in Human Platelets

**FIGURE 1:**
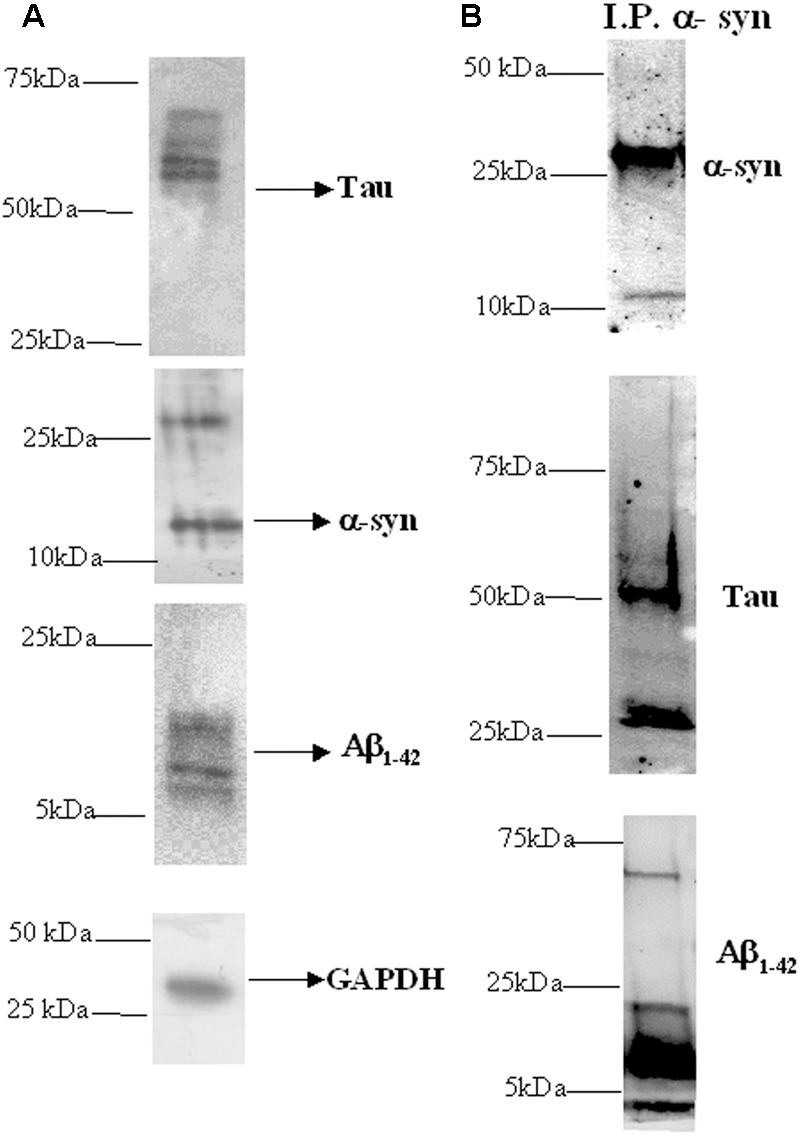
Presence of α-syn, Aβ, tau, and their heterocomplexes in platelets. **(A)** Cell lysates obtained from platelets were subjected to western blot analysis using antibody against α-syn, Aβ, or tau. GAPDH was the loading control. **(B)** Cell lysates obtained from platelets were immunoprecipitated with an anti-α-syn antibody and then immunoblotted with an α-syn, Aβ, or tau specific antibodies. One representative western blot is presented for each condition.

Western blotting analysis was used to investigate the presence of α-syn, tau and Aβ proteins in platelets isolated from healthy subjects. As depicted in **Figure 1A**, the anti-α-syn antibody produced two bands at 15 and 30 kDa (**Figure 1A**, middle panel), matching α-syn (Bartels et al., 2011), whereas the anti-tau antibody recognized three immunoreactive bands ranging between 55 and 74 kDa (Buée et al., 2000). These data confirm that platelets have detectable levels of tau and α-syn (Nakai et al., 2007; Neumann et al., 2011). Finally, the anti-Aβ antibody recognized two bands at 5 and 15 kDa proteins (**Figure 1A**, bottom panel), corresponding to Aβ monomeric and oligomer forms, respectively (Cerf et al., 2009; Nielsen et al., 2013).

Next, α-syn interaction with Aβ and tau in platelets was verified using a co-immunoprecipitation-western blotting assay (**Figure 1B**). In particular, cell lysates were immunoprecipitated using an anti-α-syn antibody, and then an immunoblot analysis was performed using an anti-tau or anti-Aβ antibody. In parallel, immunoprecipitates were blotted with the same α-syn antibody.

As expected, in α-syn immunoprecipitates (**Figure 1B**, upper panel), two bands of 15 and 30 kDa were recognized by the anti-α-syn antibody. When α-syn immunoprecipitates were probed with an anti-tau antibody (**Figure 1B**, middle panel), three immunoreactive bands ranging between 55 and 74 kDa were immunodetected (Buée et al., 2000). Additional bands, with a molecular weight lower than 50 kDa, could be related to truncated or cleaved forms of tau containing the C-terminal region (Santpere et al., 2006; Daniele et al., 2017). Finally, the Aβ immunoblotting performed on α-syn immunoprecipitates (**Figure 1B**, bottom panel) revealed multiple bands indicating monomeric and oligomeric Aβ forms (**Figure 1B**) (Nielsen et al., 2013). These data demonstrated that α-syn physically interacted with Aβ and tau in platelets, as previously demonstrated in RBCs (Daniele et al., 2017).

### α-syn Quantitative Levels in Human Platelets

**FIGURE 2:**
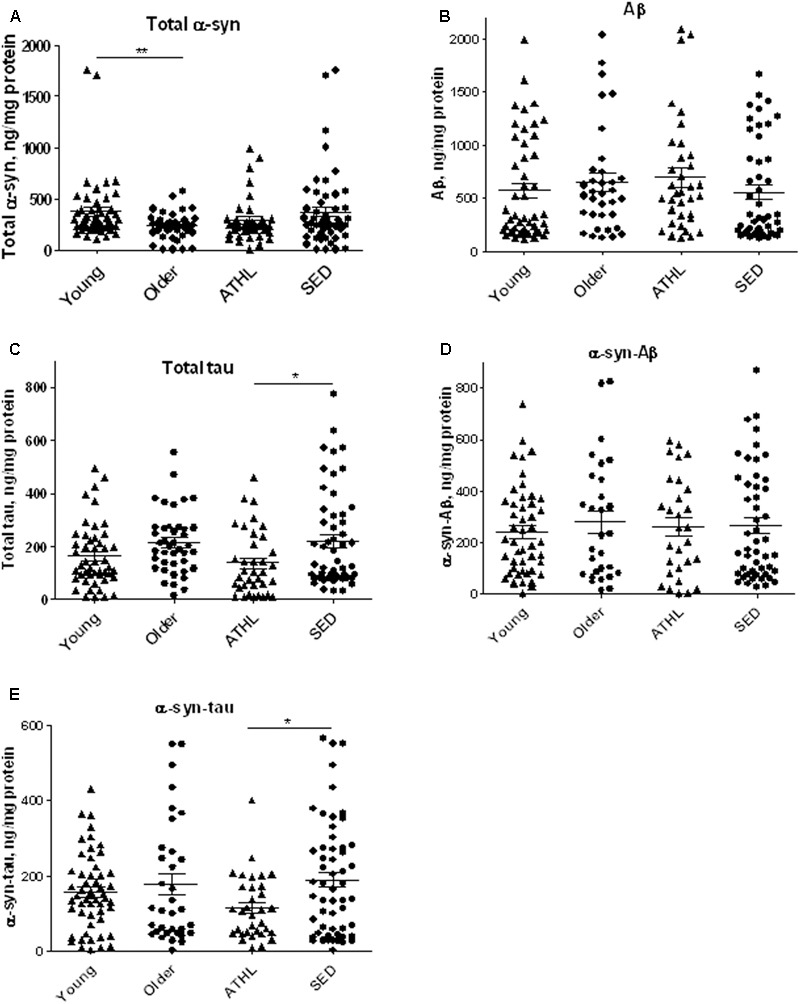
Quantitative detection of aging-related proteins in platelets. **(A–E)** Platelet levels of total α-syn **(A)**, Aβ **(B)**, tau **(C)**, α-syn/Aβ **(D)**, and α-syn/tau, **(E)** in young, older, ATHL and SED subjects (mean ± SD). Lysates obtained from platelets were subjected to specific immunoassay, as described in the Section “Materials and Methods.”. Differences between groups (i.e., young vs. older and ATHL vs. SED) were evaluated by One-way ANOVA followed by a Kruskal–Wallis *post hoc* test. *P*-values were adjusted with Sidak’s multiple comparison test: ^∗^*P* < 0.05, ^∗∗^*P* < 0.01 between the indicated subgroups.

α-syn concentrations were determined in platelets isolated from 103 healthy subjects (**Table 2**). As depicted in **Figure 2A**, older subjects presented significant lower α-syn levels with respect to young ones (young vs. older, *P* = 0.0168), suggesting that α-syn may decline with age in platelets. In contrast, no significant differences in total α-syn levels in platelets were noticed between ATHL and SED (*P* = 0.2052, **Figure 2A**), suggesting that physical exercise modulated poorly the platelet pool of α-syn.

**Table 2:** Total Oxyradical Scavenging Capacity (TOSC) values against peroxynitrite derivatives; concentrations of total α-syn, Aβ, α-syn/Aβ, tau, and α-syn/tau (as ng/mg of total protein) in the indicate subgroups.

	TOSC values peroxynitrite	Total α-syn	Aβ	α-syn/Aβ	Tau	α-syn/tau
Young subjects	18.0 ± 2.0	362 ± 275	560 ± 484	248 ± 173	170 ± 102	159 ± 99
Older subjects	17.6 ± 2.7	235 ± 132^##^	650 ± 500	280 ± 233	214 ± 107	176 ± 161
ATHL	19.3 ± 1.8	287 ± 219	697 ± 535	261 ± 197	136 ± 122	114 ± 89
SED	16.7 ± 2.1^∗∗∗^	372 ± 284	552 ± 470	267 ± 215	218 ± 182^∗^	192 ± 169^∗^

*The values are expressed as mean ± SD. ATHL, athletes, SED, sedentary. Differences between groups (i.e., young vs. older and ATHL vs. SED) were evaluated by One-way ANOVA followed by a Kruskal–Wallis *post hoc* test. *P*-values were adjusted with Sidak’s multiple comparison test. ^∗^*P* < 0.05, ^∗∗∗^*P* < 0.001 vs. the respective group of ATHL; ^##^*P* < 0.01 vs. the respective group of young subjects.*

### Aβ Quantitative Levels in Human Platelets

Aβ platelet levels showed comparable levels between young and older subjects (young vs. older, *P* = 0.5369, **Figure 2B**), as well as between ATHL and SED (ATHL vs. SED, *P* = 0.2240, **Figure 2B**).

These data suggest that age and physical exercise poorly modulate Aβ concentrations in platelets.

### Tau Quantitative Levels in Human Platelets

Similarly to what observed for Aβ, the platelet concentrations of tau showed comparable levels between young and older subjects (**Figure 2C**, young vs. older, *P* = 0.1877).

On the other hand, ATHL showed significantly lower tau levels in platelets with respect to SED in the whole population (total ATHL vs. total SED, *P* = 0.0048, **Figure 2C**). These data suggest that tau accumulation in platelets is modulated by physical exercise.

### α-syn-Tau and α-syn-Aβ Quantitative Levels in Human Platelets

Finally, the concentrations of α-syn-Aβ and α-syn-tau were measured in platelets using a previously standardized immunoenzymatic assay (Daniele et al., 2017).

As demonstrated in RBCs, platelet α-syn-Aβ or α-syn-tau levels did not differ between young and elderly subjects (*P* = 0.6734 and *P* = 0.7762, respectively, **Figures 2D,E**).

The degree of α-syn interaction with Aβ in platelets was comparable between ATHL and SED (*P* = 0.9962, **Figure 2D**). In contrast, the ATHL subjects presented significantly lower levels of platelet α-syn-tau with respect to SED, regardless of age (**Figure 2E**, ATHL vs. SED: *P* = 0.0085), suggesting that α-syn-tau levels in platelets can be modulated by physical activity.

### Correlation of ND-Related Proteins with Age

**FIGURE 3:**
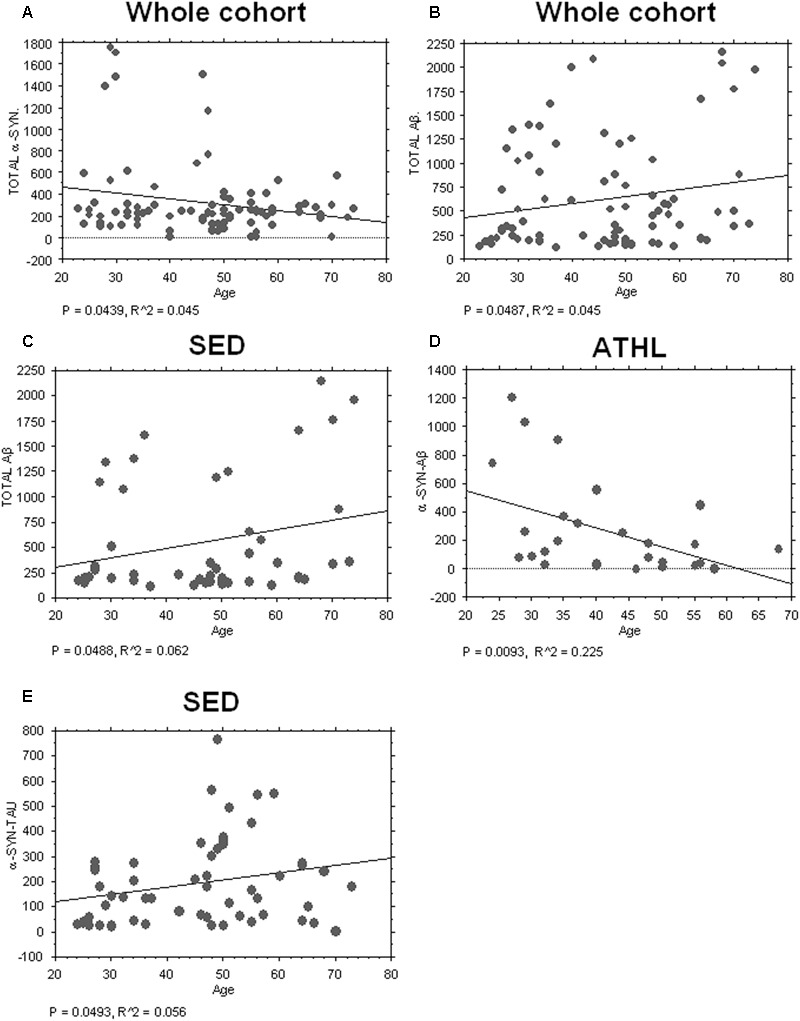
Correlation between aging-related proteins and age. **(A)** Correlation analysis between Aβ concentrations in platelets and age in whole and sedentary cohort **(B,C)** Correlation analysis between Aβ concentrations in platelets and age in whole and sedentary cohort. **(D)** Correlation analysis between α-syn/Aβ concentrations in platelets and age in the ATHL cohort. **(E)** Correlation analysis between α-syn-tau and age in the SED cohort. Correlation between variables was determined by simple linear regression analysis, using the StatView program (Abacus Concepts, Inc., SAS Institute, Cary, NC, United States). *P* and *R*^2^ values obtained for each correlation are reported in the respective panel.

Consistent with the data depicted in **Figure 2A**, an inverse correlation with age was evidenced for total α-syn in the whole cohort (total cohort: *P* = 0.0439, *R*^2^ = 0.045, **Figure 3A**).

Conversely, a significant positive correlation between Aβ levels in platelets and age was evidenced in the whole population (*P* = 0.0487, *R*^2^ = 0.038, **Figure 3B**) and in SED (*P* = 0.0488, *R*^2^ = 0.062, **Figure 3C**).

Surprisingly, significant inverse correlation between age and platelet α-syn-Aβ concentrations was found in ATHL (*P* = 0.0093, *R*^2^ = 0.225 **Figure 3D**) but not in SED (*P* = 0.1804). In contrast, α-syn-tau levels in platelets inversely correlated with age in SED (*P* = 0.0493, *R*^2^ = 0.056; **Figure 3E**) but not in ATHL (*P* = 0.1804).

### Plasma Antioxidant Capacity (AOC) in Healthy Subjects

A TOSC assay was used to determine the AOC toward peroxynitrite derivatives in plasma from healthy subjects (**Table 2**); higher mean levels from this assay are related to a better antioxidant capability (Daniele et al., 2017).

**FIGURE 4:**
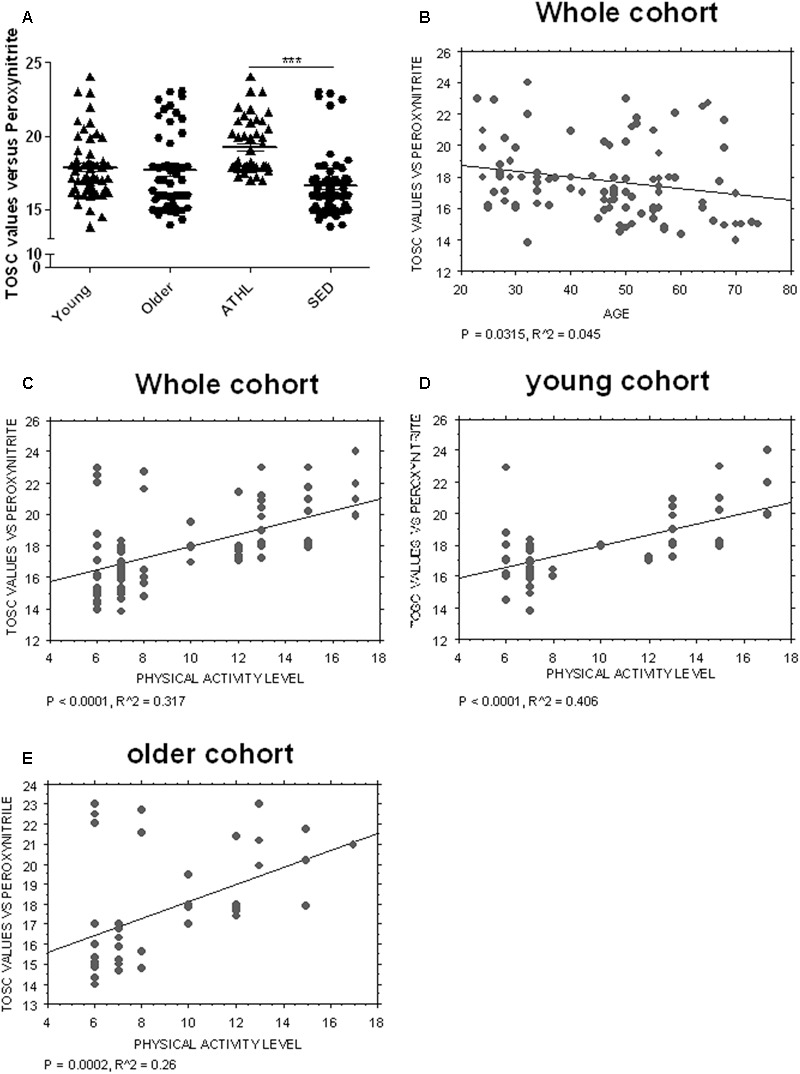
Determination of plasma AOC in human subjects. **(A)** Plasma total oxyradical scavenging capacity (TOSC) against peroxynitrate derivatives in young, older, ATHL and SED subjects (mean ± SD). Differences between groups (i.e., young vs. older and ATHL vs. SED) were evaluated by One-way ANOVA followed by a Kruskal–Wallis *post hoc* test. *P*-values were adjusted with Sidak’s multiple comparison test: ^∗∗∗^*P* < 0.001 between the indicated subgroups. **(B–E)** Correlation analysis between TOSC values against peroxynitrate derivatives and age or level of physical activity, expressed as physical activity level. Correlation between variables was determined by simple linear regression analysis, using the StatView program (Abacus Concepts, Inc., SAS Institute, Cary, NC, United States). *P* and *R*^2^ values obtained for each correlation are reported in the respective panel.

Young and older subjects showed comparable TOSC values in the total population (young vs. older, *P* = 0.3423, **Figure 4A**).

Nevertheless, TOSC values showed an inverse correlation with age in the total population (*P* = 0.0315, *R*^2^ = 0.045, **Figure 4B**).

Antioxidant capacity toward peroxynitrite derivatives was significantly higher in ATHL group with respect to SED (ATHL vs. SED, *P* < 0.0001). Consistent with these findings, peroxynitrite TOSC values showed a direct correlation with the level of physical activity (**Figures 4C–E**, total cohort: *P* < 0.0001, *R*^2^ = 0.317; young cohort: *P* < 0.0001, *R*^2^ = 0.406; older cohort: *P* = 0.0002, *R*^2^ = 0.26). These results confirm that physical exercise can enhance plasma AOC in humans (Daniele et al., 2017).

### Correlation of Aging-Related Proteins with Plasma AOC

**FIGURE 5:**
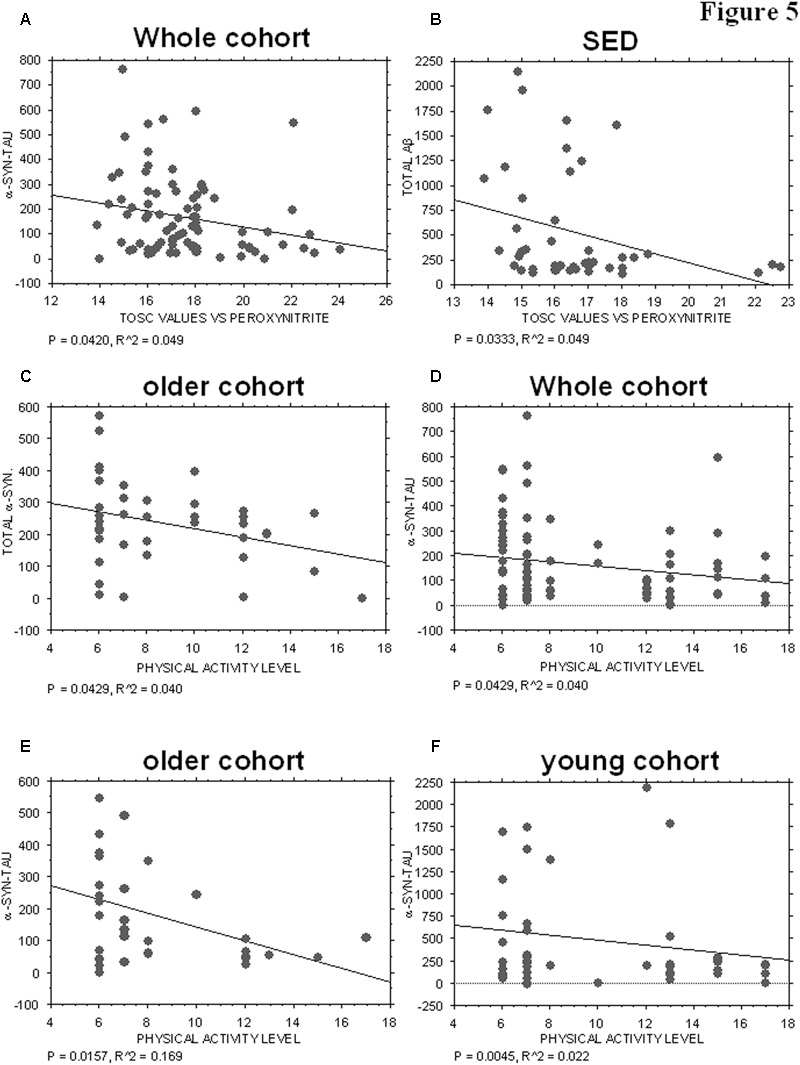
Correlation between ND-related proteins and TOSC values against peroxynitrite derivatives or physical activity level. **(A)** Correlation analysis between α-syn/tau concentrations in platelets and TOSC values against peroxynitrite derivatives in the total cohort. **(B)** Correlation analysis between Aβ concentrations in platelets and TOSC values against peroxynitrite derivatives in SED cohort. **(C)** Correlation analysis between total α-syn concentrations in platelets and physical activity level in older cohort. **(D–F)** Correlation analysis between α-syn/tau concentrations in platelets and physical activity level in total **(D)**, older **(E)** and young **(F)** cohort. Correlation between variables was determined by simple linear regression analysis, using the StatView program (Abacus Concepts, Inc., SAS Institute, Cary, NC, United States). *P* and *R*^2^ values obtained for each correlation are reported in the respective panel.

Plasma AOC toward peroxynitrite derivatives did not show any significant correlations with the platelet levels of tau (*P* = 0.3378), Aβ (*P* = 0.4195), total α-syn (*P* = 0.3704) or α-syn-Aβ (*P* = 0.8515). Interestingly, α-syn-tau levels showed an inverse correlation with TOSC values in the whole cohort (*P* = 0.0420, *R*^2^ = 0.049, **Figure 5A**), suggesting that α-syn interaction with tau in platelets can be related to plasma AOC.

Moreover, an inverse correlation was evidenced between platelet Aβ levels and TOSC values in the SED population (*P* = 0.0333, *R*^2^ = 0.099, **Figure 5B**), suggesting that Aβ accumulation in platelets may be related to plasma AOC in the presence of a low level of physical activity.

### Correlation of Aging-Related Proteins with Physical Activity

The physical activity level was not significantly related to the platelet levels of tau (whole cohort: *P* = 0.4107; older group: *P* = 0.1903), Aβ (whole cohort. *P* = 0.1694; older group: *P* = 0.7456; young group: *P* = 0.1068), or α-syn-Aβ (whole cohort: *P* = 0.9880; older group: *P* = 0.1033; young group: *P* = 0.1291). Conversely, total α-syn concentrations were inversely related to the level of physical activity in the older group (*P* = 0.0429, *R*^2^ = 0.040, **Figure 5C**), but not in the total cohort (whole cohort: *P* = 0.2688) or in the young one (*P* = 0.3072).

Interestingly, α-syn-tau concentrations showed an inverse correlation with the level of physical activity, regardless of age (**Figures 5D–F**, whole cohort: *P* = 0.0429, *R*^2^ = 0.040; older group: *P* = 0.0157, *R*^2^ = 0.169; young group: *P* = 0.0045, *R*^2^ = 0.022). These data are consistent with those reported in **Figure 3E**, and confirm that α-syn interaction with tau in platelets can be modulated negatively by physical exercise.

### Covariate Analysis

The partial correlation analysis (**Table 3**) showed that the TOSC values toward peroxynitrite derivatives were influenced primarily from age. In turn, TOSC values predicted the platelet α-syn-tau levels.

**Table 3:** Partial correlation matrix between the analyzed variables.

	Age	Physical activity level	TOSC values peroxynitrite	α-syn-Aβ	Aβ	Total α-syn	α-syn-tau	Tau
Age	1,000	-0,185	-0,001	0,070	0,181	-0,189	0,016	-0,037
15 point Borg RPE scale	-0,185	1,000	0,520	-0,007	0,329	-0,090	-0,137	-0,027
TOSC values peroxynitrite	-0,001	0,520	1,000	0,053	-0,161	-0,019	-0,029	-0,081
α-syn-Aβ	0,070	-0,007	0,053	1,000	-0,164	-0,017	-0,073	-0,129
Aβ	0,181	0,329	-0,161	-0,164	1,000	-0,057	-0,272	-0,361
Total α-syn	-0,189	-0,090	-0,019	-0,017	-0,057	1,000	-0,180	-0,165
α-syn-tau	0,016	-0,137	-0,029	-0,073	-0,272	-0,180	1,000	-0,500
Tau	-0,037	-0,027	-0,081	-0,129	-0,361	-0,165	-0,500	1,000

Interestingly, among all the analyzed variables, α-syn-Aβ concentrations in platelets were significantly decreased by physical exercise.

## Discussion

In the present paper, aging-related proteins were detected in platelets of healthy subjects (*N* = 103), and correlated to the subject AOC and to the level of physical exercise. The main findings of this work are the follows: (i) α-syn declined in older subjects, whereas Aβ directly correlated with increasing age; (ii) total α-syn and Aβ concentrations showed an inverse correlation with AOC toward peroxynitrite derivatives in elderly and sedentary subjects, respectively; (iii) tau levels decreased in athletes; (iv) α-syn directly interacted with Aβ and tau in platelets; (v) α-syn interaction with tau was inversely related to peroxynitrite AOC and to the level of physical activity. These results suggest that α-syn heterocomplexes, particularly with tau, could represent novel indicators to monitor aging-related proteins in platelets.

The loss of protein homeostasis that physiologically occur with age is characterized by the accumulation of at least one particular protein into a well-organized fibrillary structure called amyloid (Chiti and Dobson, 2006). In the case of brain aging, misfolded proteins can reach the blood compartment as the result of oligomer transfer across the blood brain barrier (DeMattos et al., 2001; Zlokovic, 2004; Eisele et al., 2010).

The accumulation and toxicity of aging-related proteins have been demonstrated to increase with raising oxidative stress in transgenic animal models and cultured cells (reviewed in Zhao and Zhao, 2013; Alavi Naini and Soussi-Yanicostas, 2015), leading a critical vicious circle.

On the other hand, several data support the notion that regular physical activity counteracts oxidative stress (Johnson and Mitchell, 2003; Lazarov et al., 2005; Radak et al., 2013) and reduces protein oligomerization in mouse brains (Brown et al., 2013; Baldacci et al., 2016; Tapia-Rojas et al., 2016; Koo et al., 2017). Recently, data on such beneficial effects have been emerging in human subjects too (DeMattos et al., 2001; Eisele et al., 2010; Brown et al., 2013).

Recently, we have investigated the influence of physical activity and antioxidant capability on the accumulation of aging-related proteins in human RBCs (Daniele et al., 2017), which represent a good peripheral model to monitor oxidative stress (Kim et al., 2008; Mohanty et al., 2014).

Herein, to unveil putative differences among the blood pools of aging-related proteins, the aforementioned parameters were examined in platelets, too.

To this purpose, a cohort of 103 healthy subjects was enrolled in the present study. Platelets were isolated from peripheral blood, and the levels of α-syn, Aβ tau, and of their heterocomplexes, were measured by immunoenzymatic assays, and related to the extent of the antioxidant capability or of physical exercise.

In our cohort, the plasma AOC was evaluated against peroxynitrite derivatives, in order to extend the data obtained toward hydroxyl and peroxyl species (Daniele et al., 2017). TOSC values confirmed an inverse correlation with age in the total population and in the sedentary cohort, as reported previously (Inal et al., 2001; Goraca, 2004). Moreover, AOC toward peroxynitrite derivatives correlated directly with the level of physical activity, regardless of subjects’ age. These results confirm that regular physical exercise can enhance plasma AOC in humans (Farah et al., 2013; Sallam and Laher, 2016; Daniele et al., 2017), and that the latter parameter is independent form the examined radical specimen.

Then, platelets were confirmed to express the age-related proteins α-syn, Aβ, tau (Cerf et al., 2009; Bartels et al., 2011; Neumann et al., 2011; Nielsen et al., 2013).

The levels of total α-syn decreased with age in the whole population, consistent with the data reported for α-syn in RBCs and plasma of healthy subjects or PD patients (Mohanty et al., 2014; Wang et al., 2015; Koehler et al., 2015; Zhao et al., 2016). Conversely, platelet Aβ levels directly correlated with age, as previously reported in RBCs (Kiko et al., 2012; Daniele et al., 2017). Considering the role of Aβ in the thrombosis formation (Shen et al., 2008), such age-dependent accumulation in human platelets could account, at least in part, for the enhanced thrombosis risk in the elderly.

Aβ concentrations inversely correlated with plasma AOC in SED, suggesting this protein as a marker of oxidative stress in peripheral cells. Consistent with this hypothesis, blood Aβ levels have been demonstrated to positively correlate with an oxidative stress marker (Kiko et al., 2012).

Then, the influence of physical exercise in ATHL and SED was examined. No significant differences between ATHL and SED were found for total α-syn levels in platelets, suggesting that platelet α-syn concentrations are poorly related to physical activity. In contrast, exercise has been shown to induce a significant reduction in the brain accumulation of α-syn (Jang et al., 2017), and α-syn levels in RBCs have been found significantly different in ATHL and SED, especially in the elderly cohort (Daniele et al., 2017). These data may suggest that the correlation between brain and peripheral fluid is cell-dependent, and, specifically, may be explained considering that α-syn is mainly expressed in RBCs (Barbour et al., 2008).

The modest influence of exercise on the platelet pool of protein was confirmed for Aβ in the present study. In contrast, significant differences have been reported in RBCs (Daniele et al., 2017) and in plasma (Brown et al., 2013) of healthy subjects. Consistent with the latter’s data, voluntary exercise has been recently shown to promote glymphatic clearance of Aβ in aged mice.

In this respect, a huge amount of literature data (reviewed in Ebrahimi et al., 2017), report the beneficial effects of physical activity on cognitive function and memory, both in transgenic mice of neurodegeneration and in patients affected by Alzheimer’s disease or Mild Cognitive Impairment. Globally, the comparison between the data obtained in this study and literature datasuggest that a different modulation of the Aβ platelet pool may occur with respect to other central or peripheral fluids. Further subjects will be needed to confirm the data on Aβ accumulation in blood cells and to understand the biological significance of these findings, both in healthy subject and in patients affects by NDs.

Interestingly, ATHL showed significantly lower tau levels in platelets with respect to SED in the whole population, suggesting that tau accumulation in platelets is modulated by physical exercise. Analogous data have been reported in RBCs of healthy subjects (Daniele et al., 2017). Similarly, aerobic exercise has been related to a reduction of phosphorylated tau protein in cerebrospinal fluid in older adults with mild cognitive impairment (Baker et al., 2015) and in transgenic mice (Ohia-Nwoko et al., 2014).

Next, the presence of α-syn associated to Aβ or tau was investigated in human platelets. α-syn was demonstrated to co-localize with tau and Aβ in platelets, as previously demonstrated in neurons or in cultured cells (Jensen et al., 1998; Masliah et al., 2001; Giasson et al., 2003; Lee et al., 2004; Mandal et al., 2006; Tsigelny et al., 2008; Badiola et al., 2011) and in RBCs (Daniele et al., 2017). As demonstrated in the latter cells, α-syn-Aβ or α-syn-tau levels in platelets did not differ between young and elderly subjects. Nevertheless, an inverse correlation between the platelet concentrations of α-syn heterocomplexes and age was found in ATHL. These data suggest that regular exercise influences the accumulation of α-syn heterocomplexes in an age-dependent manner.

α-syn-tau levels showed an inverse correlation with TOSC values and with the level of physical activity in the whole cohort, regardless of age, demonstrating that α-syn interaction with tau in platelets can be modulated by both AOC and physical exercise. Interestingly, in RBCs physical exercise has been shown to regulate α-syn interaction with Aβ rather than with tau (Daniele et al., 2017). These findings may suggest that regular exercise can exert a different modulation on α-syn heteromeric interaction, depending on the protein localization in blood.

On the light of our data, the correlation between antioxidant capability, regular activity and aging-related proteins in platelets seems to be modest with respect to that reported in brain or RBCs. This may probably account for the higher susceptibility to oxidative stress that has been reported for RBCs with respect to platelets. Further investigations could compare, in the various blood cells, the expression and activity of the enzyme related to the antioxidant system or the production and trafficking of aging-related proteins. Moreover, in such future studies the conformational state of the measured proteins will be explored.

In interpreting our data, it should be considered that these findings were obtained in platelets of a cohort of healthy subjects. The changes in the levels of aging-related proteins and α-syn heterocomplexes could not have a prognostic role in neurodegeneration, yet.

## Conclusion

The positive effect of plasma AOC and regular physical exercise on the accumulation of aging-related proteins in platelets was confirmed partially in the present paper. Most importantly, for the first time, α-syn was demonstrated to interact with Aβ and tau human platelets. In particular, this study evidenced the great modulation of α-syn interaction with tau rather than with Aβ in human platelets, probably because of the main presence of tau in these cells with respect to RBCs. These data open the way to new studies aimed at establishing the putative correlation between peripheral and central levels of α-syn heterocomplexes and their role in pathological conditions.

## Author Contributions

SD, DP, JF, CI, and LC conducted the experiments. FB, EC, and FF recruited subjects. SD, ALG, LP, EC, LC, and CG analyzed the data. SD and FF wrote the manuscript. MT, FF, GS, FG, UB and CM designed the study and provided overall supervision for the project. All authors contributed to the drafting and critical revision of the manuscript and have given final approval of the version to be published.

## Conflict of Interest Statement

The authors declare that the research was conducted in the absence of any commercial or financial relationships that could be construed as a potential conflict of interest.

## Abbreviations

AOC: antioxidant capacity
Aβ: β-amyloid_1-42_
α-syn: α-synuclein
NDs: neurodegenerative diseases
ROS: reactive oxygen species
SD: standard deviation
TBS-T: Tris buffered saline tween20

## References

[B1] Alavi Naini S. M. Soussi-Yanicostas N. (2015). Tau hyperphosphorylation and oxidative stress, a critical vicious circle in neurodegenerative tauopathies? *Oxid. Med. Cell. Longev.* 2015:151979. 10.1155/2015/151979 26576216 PMC4630413

[B2] Andersen A. D. Binzer M. Stenager E. Gramsbergen J. B. (2017). Cerebrospinal fluid biomarkers for Parkinson’s disease - a systematic review. *Acta Neurol. Scand.* 135 34–56. 10.1111/ane.12590 26991855

[B3] Badiola N. de Oliveira R. M. Herrera F. Guardia-Laguarta C. Gonçalves S. A. Pera M. (2011). Tau enhances α-synuclein aggregation and toxicity in cellular models of synucleinopathy. *PLOS ONE* 6:e26609. 10.1371/journal.pone.0026609 22039514 PMC3200341

[B4] Baker L. D. Skinner J. S. Craft S. Sink K. M. Montine T. Hansen A. (2015). Aerobic exercise reduces phosphorylated tau protein in cerebrospinal fluid in older adults with mild cognitive impairment. *Alzheimers Dementia* 11:P324 10.1016/j.jalz.2015.07.467

[B5] Baldacci F. Lista S. Garaci F. Bonuccelli U. Toschi N. Hampel H. (2016). Biomarker-guided classification scheme of neurodegenerative diseases. *J. Sport Health Sci.* 5 383–387. 10.1016/jshs.2016.08.007 PMC6188916 30356557

[B6] Barbour R. Kling K. Anderson J. P. Banducci K. Cole T. Diep L. (2008). Red blood cells are the major source of alpha-synuclein in blood. *Neurodegener. Dis.* 5 55–59. 10.1159/000112832 18182779

[B7] Bartels T. Choi J. Kim N. Selkoe D. (2011). Non-denaturing purification of alpha-Synuclein from erythrocytes. *Protoc. Exch.* 10.1038/protex.2011.254

[B8] Borg G. A. (1982). Psychophysical bases of perceived exertion. *Med. Sci. Sports Exerc.* 14 377–381. 7154893

[B9] Brown B. M. Peiffer J. J. Taddei K. Lui J. K. Laws S. M. Gupta V. B. (2013). Physical activity and amyloid-β plasma and brain levels: results from the Australian imaging, biomarkers and lifestyle study of ageing. *Mol. Psychiatry* 18 875–881. 10.1038/mp.2012.107 22889922

[B10] Buée L. Bussière T. Buée-Scherrer V. Delacourte A. Hof P. R. (2000). Tau protein isoforms, phosphorylation and role in neurodegenerative disorders. *Brain Res. Rev.* 33 95–130. 10967355 10.1016/s0165-0173(00)00019-9

[B11] Cerf E. Sarroukh R. Tamamizu-Kato S. Breydo L. Derclaye S. Dufrêne Y. F. (2009). Antiparallel beta-sheet: a signature structure of the oligomeric amyloid beta-peptide. *Biochem. J.* 421 415–423. 10.1042/BJ20090379 19435461

[B12] Chiti F. Dobson C. M. (2006). Protein misfolding, functional amyloid, and human disease. *Annu. Rev. Biochem.* 75 333–366. 10.1146/annurev.biochem.75.101304.123901 16756495

[B13] Costa B. Bendinelli S. Gabelloni P. Da Pozzo E. Daniele S. Scatena F. (2013). Human glioblastoma multiforme: p53 reactivation by a novel MDM2 inhibitor. *PLOS ONE* 8:e72281. 10.1371/journal.pone.0072281 23977270 PMC3747081

[B14] Daniele S. Pietrobono D. Fusi J. Iofrida C. Chico L. Petrozzi L. (2017). α-synuclein aggregates with β-Amyloid or tau in human red blood cells: correlation with antioxidant capability and physical exercise in human healthy subjects. *Mol. Neurobiol.* [Epub ahead of print]. 10.1007/s12035-017-0523-5 28421539

[B15] DeMattos R. B. Bales K. R. Cummins D. J. Dodart J. C. Paul S. M. Holtzman D. M. (2001). Peripheral anti-A beta antibody alters CNS and plasma A beta clearance and decreases brain A beta burden in a mouse model of Alzheimer’s disease. *Proc. Natl. Acad. Sci. U.S.A.* 98 8850–8855. 10.1073/pnas.151261398 11438712 PMC37524

[B16] Díaz-Villanueva J. F. Díaz-Molina R. García-González V. (2015). Protein folding and mechanisms of proteostasis. *Int. J. Mol. Sci.* 16 17193–17230. 10.3390/ijms160817193 26225966 PMC4581189

[B17] Ebrahimi K. Majdi A. Baghaiee B. Hosseini S. H. Sadigh-Eteghad S. (2017). Physical activity and beta-amyloid pathology in Alzheimer’s disease: a sound mind in a sound body. *EXCLI J.* 16 959–972. 10.17179/excli2017-475 28900376 PMC5579405

[B18] Eisele Y. S. Obermüller U. Heilbronner G. Baumann F. Kaeser S. A. Wolburg H. (2010). Peripherally applied Abeta-containing inoculates induce cerebral beta-amyloidosis. *Science* 330 980–982. 10.1126/science.1194516 20966215 PMC3233904

[B19] Farah C. Kleindienst A. Bolea G. Meyer G. Gayrard S. Geny B. (2013). Exercise-induced cardioprotection: a role for eNOS uncoupling and NO metabolites. *Basic Res. Cardiol.* 108:389. 10.1007/s00395-013-0389-2 24105420

[B20] Foulds P. G. Mitchell J. D. Parker A. Turner R. Green G. Diggle P. (2011). Phosphorylated α-synuclein can be detected in blood plasma and is potentially a useful biomarker for Parkinson’s disease. *FASEB J.* 25 4127–4137. 10.1096/fj.10-179192 21865317

[B21] Franzoni F. Colognato R. Galetta F. Laurenza I. Barsotti M. Di Stefano R. (2006). An in vitro study on the free radical scavenging capacity of ergothioneine: comparison with reduced glutathione, uric acid and trolox. *Biomed. Pharmacother.* 60 453–457. 10.1016/j.biopha.2006.07.015 16930933

[B22] Franzoni F. Ghiadoni L. Galetta F. Plantinga Y. Lubrano V. Huang Y. (2005). Physical activity, plasma antioxidant capacity, and endothelium-dependent vasodilation in young and older men. *Am. J. Hypertens.* 18 510–516. 10.1016/j.amjhyper.2004.11.006 15831361

[B23] Giacomelli C. Daniele S. Martini C. (2017). Potential biomarkers and novel pharmacological targets in protein aggregation-related neurodegenerative diseases. *Biochem. Pharmacol.* 131 1–15. 10.1016/j.bcp.2017.01.017 28159621

[B24] Giasson B. I. Forman M. S. Higuchi M. Golbe L. I. Graves C. L. Kotzbauer P. T. (2003). Initiation and synergistic fibrillization of tau and alpha-synuclein. *Science* 300 636–640. 10.1126/science.1082324 12714745

[B25] Goedert M. (2015). Alzheimer’s and Parkinson’s diseases: the prion concept in relation to assembled Aβ, tau, and α-synuclein. *Science* 349:1255555. 10.1126/science.1255555 26250687

[B26] Goraca A. (2004). Assessment of total antioxidant capacity in human plasma. *Folia Med.* 46 16–21. 15962810

[B27] Hamer P. Slocombe B. (1997). The psychophysical and heart rate relationship between treadmill and deep-water running. *Aust. J. Physiother.* 43 265–271. 11676696 10.1016/s0004-9514(14)60415-3

[B28] Hinault M. P. Ben-Zvi A. Goloubinoff P. (2006). Chaperones and proteases: cellular fold-controlling factors of proteins in neurodegenerative diseases and aging. *J. Mol. Neurosci.* 30 249–265. 17401151 10.1385/JMN:30:3:249

[B29] Inal M. E. Kanbak G. Sunal E. (2001). Antioxidant enzyme activities and malondialdehyde levels related to aging. *Clin. Chim. Acta* 305 75–80. 11249925 10.1016/s0009-8981(00)00422-8

[B30] Jang Y. Koo J. H. Kwon I. Kang E. B. Um H. S. Soya H. (2017). Neuroprotective effects of endurance exercise against neuroinflammation in MPTP-induced Parkinson’s disease mice. *Brain Res.* 1655 186–193. 10.1016/j.brainres.2016.10.029 27816415

[B31] Jensen P. H. Nielsen M. S. Jakes R. Dotti C. G. Goedert M. (1998). Binding of alpha-synuclein to brain vesicles is abolished by familial Parkinson’s disease mutation. *J. Biol. Chem.* 273 26292–26294. 9756856 10.1074/jbc.273.41.26292

[B32] Johnson R. A. Mitchell G. S. (2003). Exercise-induced changes in hippocampal brain-derived neurotrophic factor and neurotrophin-3: effects of rat strain. *Brain Res.* 983 108–114. 12914971 10.1016/s0006-8993(03)03039-7

[B33] Jucker M. Walker L. C. (2013). Self-propagation of pathogenic protein aggregates in neurodegenerative diseases. *Nature* 501 45–51. 10.1038/nature12481 24005412 PMC3963807

[B34] Kiko T. Nakagawa K. Satoh A. Tsuduki T. Furukawa K. Arai H. (2012). Amyloid β levels in human red blood cells. *PLOS ONE* 7:e49620. 10.1371/journal.pone.0049620 23166730 PMC3499416

[B35] Kim Y. K. Kwon E. H. Kim D. H. Won D. I. Shin S. Suh J. S. (2008). Susceptibility of oxidative stress on red blood cells exposed to gamma rays: hemorheological evaluation. *Clin. Hemorheol. Microcirc.* 40 315–324. 19126995

[B36] Koehler N. K. Stransky E. Meyer M. Gaertner S. Shing M. Schnaidt M. (2015). Alpha-synuclein levels in blood plasma decline with healthy aging. *PLOS ONE* 10:e0123444. 10.1371/journal.pone.0123444 25844871 PMC4386828

[B37] Koo J. H. Kang E. B. Oh Y. S. Yang D. S. Cho J. Y. (2017). Treadmill exercise decreases amyloid-β burden possibly via activation of SIRT-1 signaling in a mouse model of Alzheimer’s disease. *Exp. Neurol.* 288 142–152. 10.1016/j.expneurol.2016.11.014 27889467

[B38] Labbadia J. Morimoto R. I. (2015). The biology of proteostasis in aging and disease. *Annu. Rev. Biochem.* 84 435–464. 10.1146/annurev-biochem-060614-033955 25784053 PMC4539002

[B39] Lazarov O. Robinson J. Tang Y. P. Hairston I. S. Korade-Mirnics Z. Lee V. M. (2005). Environmental enrichment reduces Abeta levels and amyloid deposition in transgenic mice. *Cell* 120 701–713. 10.1016/j.cell.2005.01.015 15766532

[B40] Lee V. M. Giasson B. I. Trojanowski J. Q. (2004). More than just two peas in a pod: common amyloidogenic properties of tau and alpha-synuclein in neurodegenerative diseases. *Trends Neurosci.* 27 129–134. 10.1016/j.tins.2004.01.007 15036877

[B41] Lindner A. B. Demarez A. (2009). Protein aggregation as a paradigm of aging. *Biochim. Biophys. Acta* 1790 980–996. 10.1016/j.bbagen.2009.06.005 19527771

[B42] Mandal P. K. Pettegrew J. W. Masliah E. Hamilton R. L. Mandal R. (2006). Interaction between Abeta peptide and alpha synuclein: molecular mechanisms in overlapping pathology of Alzheimer’s and Parkinson’s in dementia with Lewy body disease. *Neurochem. Res.* 31 1153–1162. 10.1007/s11064-006-9140-9 16947080

[B43] Masliah E. Rockenstein E. Veinbergs I. Sagara Y. Mallory M. Hashimoto M. (2001). Beta-amyloid peptides enhance alpha-synuclein accumulation and neuronal deficits in a transgenic mouse model linking Alzheimer’s disease and Parkinson’s disease. *Proc. Natl. Acad. Sci. U.S.A.* 98 12245–12250. 10.1073/pnas.211412398 11572944 PMC59799

[B44] Mohanty J. G. Nagababu E. Rifkind J. M. (2014). Red blood cell oxidative stress impairs oxygen delivery and induces red blood cell aging. *Front. Physiol.* 5:84. 10.3389/fphys.2014.00084 24616707 PMC3937982

[B45] Morimoto R. I. Cuervo A. M. (2014). Proteostasis and the aging proteome in health and disease. *J. Gerontol. A Biol. Sci. Med. Sci.* 69 S33–S38. 10.1093/gerona/glu049 24833584 PMC4022129

[B46] Nakai M. Fujita M. Waragai M. Sugama S. Wei J. Akatsu H. (2007). Expression of alpha-synuclein, a presynaptic protein implicated in Parkinson’s disease, in erythropoietic lineage. *Biochem. Biophys. Res. Commun.* 358 104–110. 10.1016/j.bbrc.2007.04.108 17475220

[B47] Neumann K. Farías G. Slachevsky A. Perez P. Maccioni R. B. (2011). Human platelets tau: a potential peripheral marker for Alzheimer’s disease. *J. Alzheimers Dis.* 25 103–109. 10.3233/JAD-2011-101641 21368381

[B48] Nielsen H. M. Ek D. Avdic U. Orbjörn C. Hansson O. Veerhuis R. (2013). NG2 cells, a new trail for Alzheimer’s disease mechanisms? *Acta Neuropathol. Commun.* 1:7. 10.1186/2051-5960-1-7 24252600 PMC4046664

[B49] Ohia-Nwoko O. Montazari S. Lau Y. S. Eriksen J. L. (2014). Long-term treadmill exercise attenuates tau pathology in P301S tau transgenic mice. *Mol. Neurodegener.* 9:54. 10.1186/1750-1326-9-54 25432085 PMC4280713

[B50] Pandey K. B. Rizvi S. I. (2010). Markers of oxidative stress in erythrocytes and plasma during aging in humans. *Oxid. Med. Cell. Longev.* 3 2–12. 10.4161/oxim.3.1.10476 20716923 PMC2835884

[B51] Parnetti L. Castrioto A. Chiasserini D. Persichetti E. Tambasco N. El-Agnaf O. (2013). Cerebrospinal fluid biomarkers in Parkinson disease. *Nat. Rev. Neurol.* 9 131–140. 10.1038/nrneurol.2013.10 23419373

[B52] Pesini P. Pérez-Grijalba V. Monleón I. Boada M. Tárraga L. Martínez-Lage P. (2012). Reliable measurements of the β-Amyloid pool in blood could help in the early diagnosis of AD. *Int. J. Alzheimers Dis.* 2012:604141. 10.1155/2012/604141 22957297 PMC3431090

[B53] Radak T. Marton O. Nagy E. Koltai E. Goto S. (2013). The complex role of physical exercise and reactive oxygen species on brain. *J. Sport Health Sci.* 2 87–93. 10.1016/j.jshs.2013.04.001

[B54] Radak Z. Suzuki K. Higuchi M. Balogh L. Boldogh I. Koltai E. (2016). Physical exercise, reactive oxygen species and neuroprotection. *Free Radic. Biol. Med.* 98 187–196. 10.1016/j.freeradbiomed.2016.01.024 26828019

[B55] Regoli F. Winston G. W. (1999). Quantification of total oxidant scavenging capacity of antioxidants for peroxynitrite, peroxyl radicals, and hydroxyl radicals. *Toxicol. Appl. Pharmacol.* 156 96–105. 10.1006/taap.1999.8637 10198274

[B56] Sallam N. Laher I. (2016). Exercise modulates oxidative stress and inflammation in aging and cardiovascular diseases. *Oxid. Med. Cell. Longev.* 2016:7239639. 10.1155/2016/7239639 26823952 PMC4707375

[B57] Santpere G. Puig B. Ferrer I. (2006). Low molecular weight species of tau in Alzheimer’s disease are dependent on tau phosphorylation sites but not on delayed post-mortem delay in tissue processing. *Neurosci. Lett.* 399 106–110. 10.1016/j.neulet.2006.01.036 16488541

[B58] Sengupta U. Guerrero-Muñoz M. J. Castillo-Carranza D. L. Lasagna-Reeves C. A. Gerson J. E. Paulucci-Holthauzen A. A. (2015). Pathological interface between oligomeric alpha-synuclein and tau in synucleinopathies. *Biol. Psychiatry* 78 672–683. 10.1016/j.biopsych.2014.12.019 25676491

[B59] Shen M. Y. Hsiao G. Fong T. H. Chou D. S. Sheu J. R. (2008). Expression of amyloid beta peptide in human platelets: pivotal role of the phospholipase Cgamma2-protein kinase C pathway in platelet activation. *Pharmacol. Res.* 57 151–158. 10.1016/j.phrs.2008.01.004 18313326

[B60] Snead D. Eliezer D. (2014). Alpha-synuclein function and dysfunction on cellular membranes. *Exp. Neurobiol.* 23 292–313. 10.5607/en.2014.23.4.292 25548530 PMC4276801

[B61] Tan C. C. Yu J. T. Tan M. S. Jiang T. Zhu X. C. Tan L. (2014). Autophagy in aging and neurodegenerative diseases: implications for pathogenesis and therapy. *Neurobiol. Aging* 35 941–957. 10.1016/j.neurobiolaging.2013.11.019 24360503

[B62] Tapia-Rojas C. Aranguiz F. Varela-Nallar L. Inestrosa N. C. (2016). Voluntary running Attenuates memory loss, decreases neuropathological changes and Induces neurogenesis in a mouse model of Alzheimer’s Disease. *Brain Pathol.* 26 62–74. 10.1111/bpa.12255 25763997 PMC8029165

[B63] Tokuda T. Qureshi M. M. Ardah M. T. Varghese S. Shehab S. A. Kasai T. (2010). Detection of elevated levels of α-synuclein oligomers in CSF from patients with Parkinson disease. *Neurology* 75 1766–1772. 10.1212/WNL.0b013e3181fd613b 20962290

[B64] Tsigelny I. F. Crews L. Desplats P. Shaked G. M. Sharikov Y. Mizuno H. (2008). Mechanisms of hybrid oligomer formation in the pathogenesis of combined Alzheimer’s and Parkinson’s diseases. *PLOS ONE* 3:e3135. 10.1371/journal.pone.0003135 18769546 PMC2519786

[B65] Veitinger M. Varga B. Guterres S. B. Zellner M. (2014). Platelets, a reliable source for peripheral Alzheimer’s disease biomarkers? *Acta Neuropathol. Commun.* 2:65. 10.1186/2051-5960-2-65 24934666 PMC4229876

[B66] Voss M. W. Vivar C. Kramer A. F. van Praag H. (2013). Bridging animal and human models of exercise-induced brain plasticity. *Trends Cogn. Sci.* 17 525–544. 10.1016/j.tics.2013.08.001 24029446 PMC4565723

[B67] Wang X. Yu S. Li F. Feng T. (2015). Detection of α-synuclein oligomers in red blood cells as a potential biomarker of Parkinson’s disease. *Neurosci. Lett.* 599 115–119. 10.1016/j.neulet.2015.05.030 25998655

[B68] Whaley M. H. Brubaker P. H. Otto R. M. Armstrong L. E. (2006). *Medicine ACoS Guidelines for Exercise Testing and Prescription* 7th Edn. Philadelphia, PA: Lippincott Williams & Wilkins.

[B69] Winston G. W. Regoli F. Dugas A. J. Fong J. H. Blanchard K. A. (1998). A rapid gas chromatographic assay for determining oxyradical scavenging capacity of antioxidants and biological fluids. *Free Radic. Biol. Med.* 24 480–493. 9438561 10.1016/s0891-5849(97)00277-3

[B70] Xiang W. Menges S. Schlachetzki J. C. Meixner H. Hoffmann A. C. Schlötzer-Schrehardt U. (2015). Posttranslational modification and mutation of histidine 50 trigger alpha synuclein aggregation and toxicity. *Mol. Neurodegener.* 10:8. 10.1186/s13024-015-0004-0 25886189 PMC4365527

[B71] Zhao H. Q. Li F. F. Wang Z. Wang X. M. Feng T. (2016). A comparative study of the amount of α-synuclein in ischemic stroke and Parkinson’s disease. *Neurol. Sci.* 37 749–754. 10.1007/s10072-016-2485-1 26829934

[B72] Zhao Y. Zhao B. (2013). Oxidative stress and the pathogenesis of Alzheimer’s disease. *Oxid. Med. Cell. Longev.* 2013:316523. 10.1155/2013/316523 23983897 PMC3745981

[B73] Zlokovic B. V. (2004). Clearing amyloid through the blood-brain barrier. *J. Neurochem.* 89 807–811. 10.1111/j.1471-4159.2004.02385.x 15140180

